# Natural Tooth Pontic Using Recent Adhesive Technologies: An Esthetic and Minimally Invasive Prosthetic Solution

**DOI:** 10.1155/2020/7619715

**Published:** 2020-02-05

**Authors:** Davide Augusti, Gabriele Augusti, Andrei Ionescu, Eugenio Brambilla, Dino Re

**Affiliations:** Department of Biomedical, Surgical and Dental Sciences, University of Milan, Italy

## Abstract

An implant-supported crown represents an established and validated option for single-tooth replacement; however, a restorative solution should be selected according to a wide number of factors including patient's desire, expectations, specific clinical conditions, and financial possibilities. The aim of this case report is to describe a conservative rehabilitation strategy for the replacement of a periodontally compromised mandibular incisor: the extracted natural tooth was used as a pontic bonded to adjacent elements with polyethylene fiber and resin composite. This way, a chairside fabrication of a resin-bonded fiber-reinforced prosthesis is possible, using the patient's own tooth. After showing a satisfactory functional and esthetic result, advantages and pitfalls of this technique along with available data on the literature regarding the natural tooth pontic are addressed. Both patients and clinicians should be aware of minimally invasive, successful solutions for single-tooth replacement; whether indicated or necessary, the natural tooth pontic technique leaves open other treatment options for the future.

## 1. Introduction

The replacement of a single missing or failing tooth presents one of the greatest challenges in restorative dentistry, especially when the esthetic zone is considered [1]. Nowadays, the main available treatment options to this clinical problem include the use of traditional fixed dental prostheses (FDPs), implant-supported single crowns (SCI), and resin-bonded FDPs (RBFPDs) [2, 3].

Resin-bonded fixed partial dentures (RBFPDs) were first introduced into dentistry in the 1970s [4]: their primary objective was to splint periodontally compromised teeth along with substitution of one or more missing anterior teeth. The application of RBFPDs was extended to posterior areas about ten years later [5]. Compared to implant-supported solutions, RBFPDs are linked to short treatment times and lower postoperative morbidity and costs; surgical procedures are also avoided [6]. With respect to traditional fixed prostheses, RBFPDs can be delivered by minimal or no preparation of natural abutments: this way, an excellent preservation of tooth structure (with reduction of pulpal morbidity) might be achieved [6–8]. The expected survival for indirect (lab-fabricated) RBFPD is relatively high [9]: according to a recent study by Thoma et al., a 91.4% and 82.9% rates were reported after 5 and 10 years of observation, respectively; overcoming complications were mainly related to debonding (15%) and chipping of veneering material (4%). According to a recent systematic review, clinicians should consider using RBFPDs more often because their clinical performance is similar to those of conventional FPDs and implant-supported crowns [10].

Historically, cast RBFPDs were produced exclusively using noble metals like high-gold alloys [5]; nowadays, a wide range of new materials is available: fiber-reinforced composites [11], ceramics with a high content of glass particles (i.e., lithium disilicate, glass-infiltrated zirconia, or alumina) [7, 12], or high-strength ceramics (densely sintered zirconia/alumina polycristal) might be used as frameworks for subsequent veneering or to fabricate monolithic restorations [8, 13].

Fiber-reinforced resin composites (FRC) have been widely adopted in dentistry [14], as direct materials to fabricate periodontal, posttraumatic, or orthodontic splints to stabilize teeth, and for indirect restorative purposes as well. FRC materials consist of glass, carbon, or polyethylene fibers contained within a resin matrix; the type of fiber, its architecture (i.e., spatial arrangement), and the quality of the fiber/matrix coupling determine the mechanical properties of the material [14, 15]. Laboratory studies have shown that FRC materials exhibit a flexural strength that is greater than unreinforced flow or traditional composite materials [15, 16]. By using FRCs, both FDP and RBFDP frameworks can be realized in a minimal invasive fashion, utilizing combinations of various kinds of adhering and retentive elements (like surface bonding wings on anterior areas of the mouth) [11]. A direct, intraoral fabrication of an anterior resin-bonded FRC prosthesis is possible using prefabricated pontics, a denture tooth, or an extracted natural tooth [17].

The immediate bonding of a natural tooth to adjacent elements presents a low-cost alternative for direct tooth replacement [6, 17–19]; this technique enables the original tooth anatomy to be replaced, providing excellent function and esthetics (size, shape, and color) at the same time. Use of patient's own tooth as pontic represents a conservative restorative solution with no laboratory procedures involved; it is well-suited for patients who ask for an immediate replacement of a hopeless tooth in the esthetic zone [20, 21] and are not candidates for implant therapy. The use of a natural tooth as pontic (NTP) provides promising results by means of a combined application of fiber-reinforced materials and adhesive technologies.

The aim of this study is to describe a conservative rehabilitation strategy for the replacement of a periodontally compromised mandibular incisor: the extracted natural tooth was used as pontic, precisely repositioned with a customized index, and finally bonded to adjacent elements with polyethylene ribbon and resin composite. After case illustration, an analysis of available data in the literature related to NTP and associated outcomes will be addressed.

## 2. Case Presentation

### 2.1. Patient Presentation and Chief Complaint

**Figure 1:**
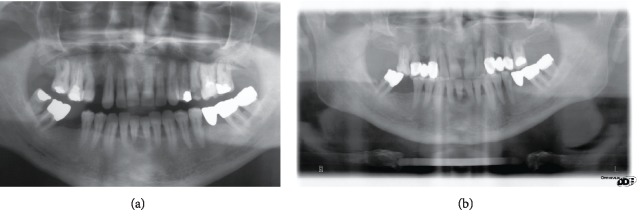
Panoramic radiographs of the patient exposed at year 2008 (a) and 2017 (b): loss of teeth and periodontal breakdown are visible.

A male, 55-year-old Caucasian patient presented at our private practice seeking treatment for increased tooth mobility at the lower right central incisor (tooth number 4.1). The medical history revealed a longstanding, drug-controlled type II diabetes mellitus and single-episode acute pancreatitis. The patient was a nonsmoker and had received some previous treatments in our office in the past; however, he demonstrated poor adherence to follow-up visits and maintenance care. Two panoramic radiographs (Figures 1(a) and 1(b)) were available to evaluate intraoral changes developed during a timespan of approximately ten years (2008-2017). An overall periodontal breakdown was noted with horizontal bone loss, especially at upper and lower anterior areas; upper first premolars and second molars had been extracted, and traditional fixed dental prostheses had been used to replace elements 1.4 and 2.4. In the lower arch, angular bony defects were present at mesial surfaces of teeth 4.7, 4.3, and 3.7/occlusion/overjet/overbite.

**Figure 2:**
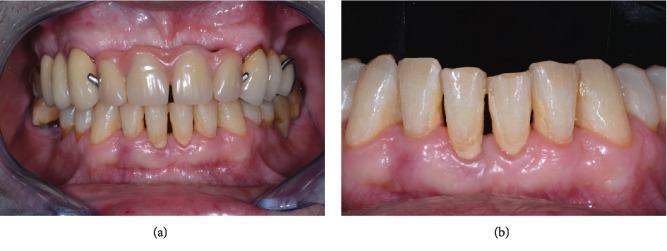
Clinical presentation of the patient at initial examination: (a) full-mouth and (b) frontal lower incisors views.

**Figure 3:**
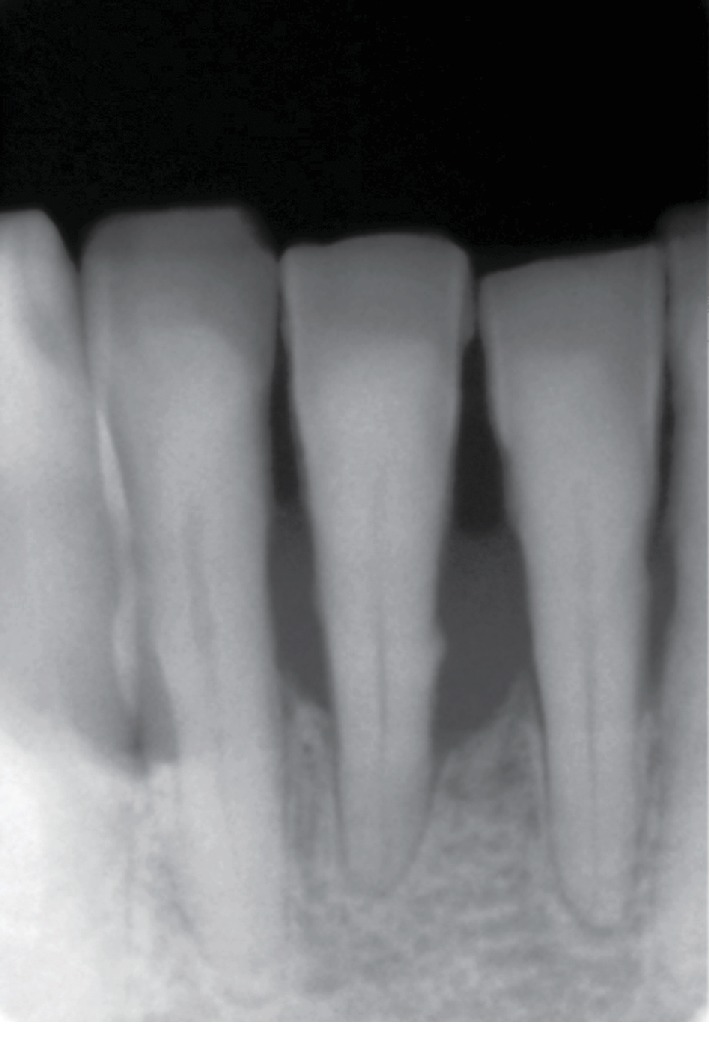
A poor periodontal prognosis was assigned to the lower central incisor, showing more than 80% of bone support loss.

Clinically, tooth 4.1 exhibited a deep probing pocket depth (PPD > 10 mm) on both mesial and distal sides, in association with class 2 mobility according to Miller's index (i.e., the tooth is held between the metallic handles of two instruments and moved in the buccolingual or buccopalatal direction; the moved distance is visually estimated by the person carrying out the examination: a score of 2 means a detectable horizontal mobility superior to 1 mm) [22]. Gingival margin inflammation and recession (2 mm) and loss of interdental papilla between lower incisors and supragingival calculus were noted (Figures 2(a) and 2(b)). A periapical radiograph revealed more than 80% of bone support loss and subgingival calculus along the root surface (Figure 3). A poor periodontal prognosis was assigned to the tooth.

### 2.2. Treatment Plan

During consultation, clinical problems related to the tooth and side effects associated to extraction without replacement were explained to the patient; his main desire was to preserve function and receiving at the same time a cost-effective treatment. In addition, the immediate prosthetic replacement at the stage of surgery was not a priority for the patient: an agreement for a delayed approach was reached (see next). Due to systemic and dental local conditions, a surgical plan based on implant-supported prosthesis (either immediate or delayed) was discarded; adjacent teeth were not affordable abutments for a traditional, fixed, definitive dental bridge.

Despite a poor periodontal prognosis, the color, shape, and position of the lower central incisor were considered acceptable (i.e., good esthetic integration with adjacent teeth). According to the above considerations, a chairside fiber-reinforced composite bridge using the patient's own natural tooth as pontic was the selected therapeutic approach; informed consent was obtained.

### 2.3. Silicone Index and Tooth Extraction

**Figure 4:**
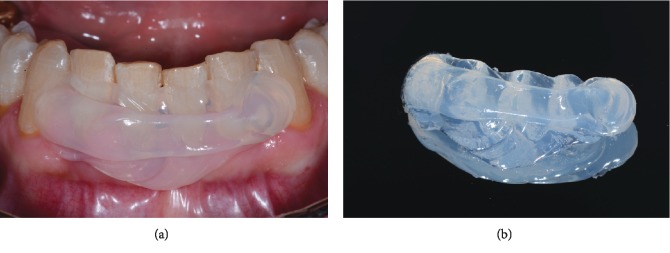
(a) Recording of the lower central incisor position using a transparent silicone index; (b) extraoral view of the matrix.

A preliminary prophylaxis for supra- and subgingival calculus debridement was scheduled: a single session, full-mouth disinfection with mechanical scaling and root planing was carried out one week before extraction. The spatial relationship of the hopeless tooth with adjacent incisors was recorded: a silicone-based, medium-consistency bite registration material (Glassbite® Clear, 80shore; Detax, Ettlingen, Germany) was directly applied on the lower anterior teeth (from canine to canine) and allowed to set (Figure 4(a)). A transparent rigid matrix was finally obtained for accurate repositioning of the lower central incisor after extraction (Figure 4(b)). Customized resin splints have been also suggested for the same purpose [18, 23].

**Figure 5:**
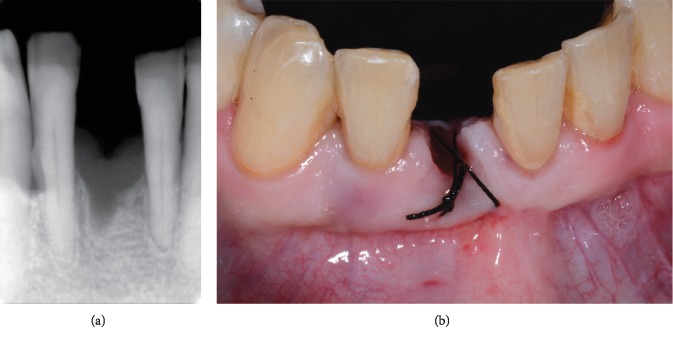
(a) The hopeless tooth was extracted; (b) a cross-suture was useful for clot stabilization.

Under local anesthesia, the lower central incisor was atraumatically extracted with forceps; the alveolar socket was carefully debrided/degranulated (with the aid of hand excavators) and finally rinsed with saline solution. 4-0 silk crossed sutures were used for gingival and clot stabilization; a postextraction radiograph was exposed (Figures 5(a) and 5(b)). After surgery, initial soft tissue closure and remodeling were deemed necessary for two main reasons: (1) an unpredictable socket healing due to systemic conditions of the patient, suboptimal hygiene control, and periodontal disease affecting the tooth [24]and (2) to provide a more accurate trimming of the extracted crown-root complex following the initial apical repositioning of soft tissues. In the meantime, proper treatment and storage of the extracted tooth was carried out.

### 2.4. Treatment of the Extracted Tooth and Storage

A traditional endodontic access cavity was prepared; coronal pulp tissue was mechanically removed and chemically dissolved from the chamber to avoid later discoloration through decomposing of organic remnants. The root canal was instrumented using stainless steel manual K-files (up to ISO size #25), along with 5.25% NaOCL and EDTA irrigations. To prevent dehydration, the tooth was kept in saline solution until any further manipulation.

### 2.5. Try-In

**Figure 6:**
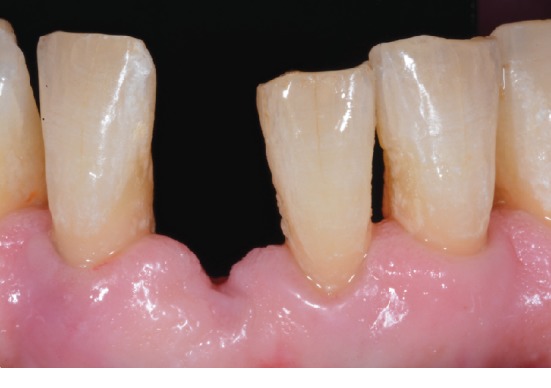
Soft tissue healing seven weeks after tooth extraction.

**Figure 7:**
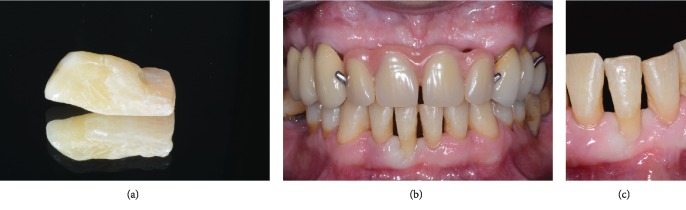
(a) Partial resection of the root portion; clinical try-in of the extracted and trimmed tooth revealed slight compression of gingival tissues (b, c).

**Figure 8:**
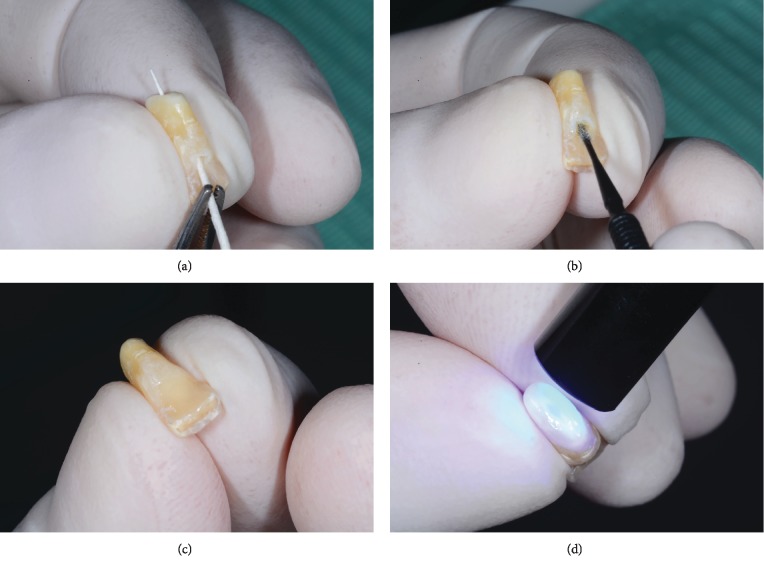
(a) After preliminary etching and rinsing, the tooth canal and pulp chamber were carefully dried with paper points. (b) A single-component, light-curing universal adhesive was applied using a narrow microbrush. (c) Flowable resin composite was used for sealing of both the access cavity and the root-end preparation. (d) Light-curing activation at the pontic surface.

Seven weeks after the extraction procedure, an uneventful healing of soft tissues was confirmed (Figure 6). The root of the extracted tooth was shortened (Figure 7(a)); resection was carried out to produce slight compression and transient ischemia (within three minutes) to the surrounding gingiva (Figures 7(b) and 7(c)). The root canal was irrigated and dried with standardized paper points; a universal adhesive (Scotchbond™ Universal Adhesive, 3M ESPE) was applied with a microbrush along the root canal/pulp chamber walls and photoactivated. Flowable resin composite (Clearfil™ Majesty ES Flow, Kuraray Noritake Dental Inc.) was used for sealing of both the access cavity and the root-end preparation; complete light-curing polymerization of 20 s per surface was performed (light unit Bluephase C8, Ivoclar Vivadent). The sealed root-end was shaped to obtain an ovate pontic configuration: the surface was finished to have a smooth and convex contour; final polishing was carried out using pumice paste (coarse/fine grit, Kerr). Steps for the preparation of the extracted tooth are illustrated in Figure 8.

### 2.6. Splinting of the Natural Tooth Pontic

**Figure 9:**
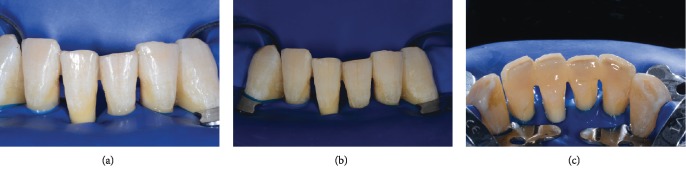
(a) Vestibular view of completed restoration under rubber dam isolation using (a) standardized flash-ring illumination and (b) cross-polarized exposures. (c) Lingual view showing resin composite fully covering the polyethylene fiber.

**Figure 10:**
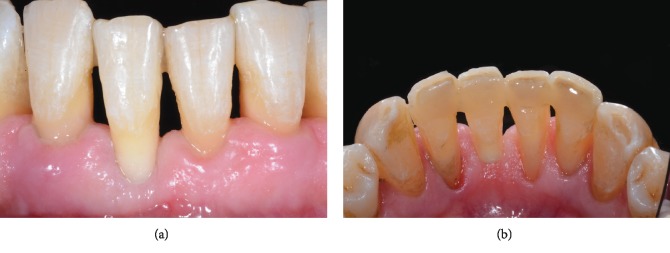
Intraoral (a) vestibular and (b) lingual views of the restoration immediately after rubber dam removal.

**Figure 11:**
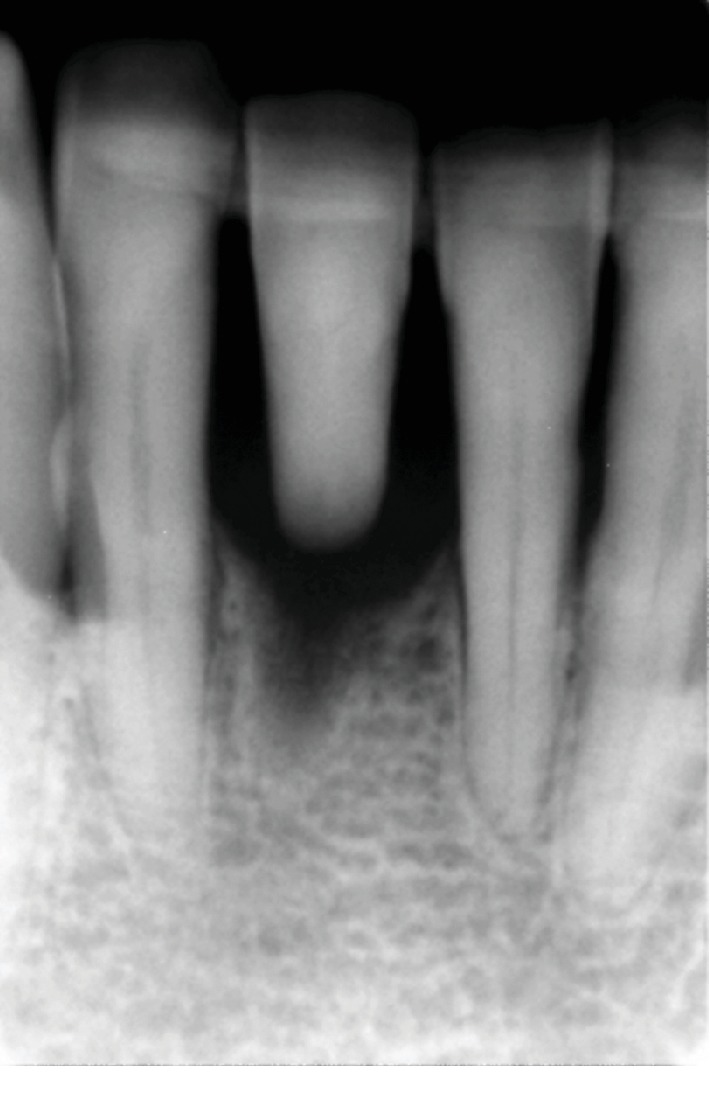
Periapical radiograph showing the natural tooth pontic bonded to adjacent elements; the radiopaque fiber extending to all lower incisors can be appreciated.

Proper seating and proximal contacts of the natural tooth pontic were checked, along with the correct shade and texture integration before rubber dam application. The length of the polyethylene fiber (Ribbond™ Ultra, 2 mm width; Ribbond Inc., Seattle, WA, US) required for splinting was measured (with the aid of the previously fabricated silicone index) and cut: all four lower incisors were included. A relative coronal insertion of the fiber was selected to ensure adequate interdental cleaning (with interproximal brushes) of periodontally compromised teeth. Following rubber dam isolation, a superficial groove was prepared on the lingual surfaces of the incisors (coronal third) to inlay the splint (Ribbond™ Ultra thickness: 0.12 mm); a corresponding slot preparation was carried out on the natural tooth pontic at the same level of adjacent teeth. The lingual and proximal surfaces were conditioned with 35% phosphoric acid, rinsed, and dried thoroughly; a single-bottle, multipurpose, light-curing universal adhesive resin ((Scotchbond™ Universal Adhesive, 3M ESPE) was applied to etched preparations according to manufacturer instructions and light cured for 40 seconds. A thin layer of flowable composite resin (Clearfil™ Majesty ES Flow, Kuraray Noritake Dental Inc.) was applied to the grooves and proximal surfaces of adjacent teeth. Just before placement, the fiber was impregnated with the adhesive bonding agent; then, it was precisely adapted to the preparations with hand instrument—in order to obtain an excellent fit into the slots—and light cured from multiple directions (20 s per surface). The polyethylene fiber is adapted easily to dental contours and can be manipulated during the bonding process; a passive application prevented unintentional orthodontic movement of involved teeth. During flow and fiber placement, the pontic was maintained in a correct spatial relationship with adjacent teeth using the silicone index. A direct composite resin was finally placed and photopolymerized to completely cover the fiber and restore the lingual anatomy of the incisors (Figure 9). All margins were carefully refined and polished using silicon carbide brushes (Jiffy Brushes, Ultradent), until smooth lingual surfaces were obtained. The completed restoration is shown at rubber dam removal (Figures 10(a) and 10(b)); centric and excursive occlusion was verified with articulating papers; premature contacts were eliminated. A final radiograph of the bonded natural tooth pontic was exposed (Figure 11); oral hygiene instructions were given to the patient for proper interdental and under-the-pontic cleanings.

### 2.7. Follow-Up

**Figure 12:**
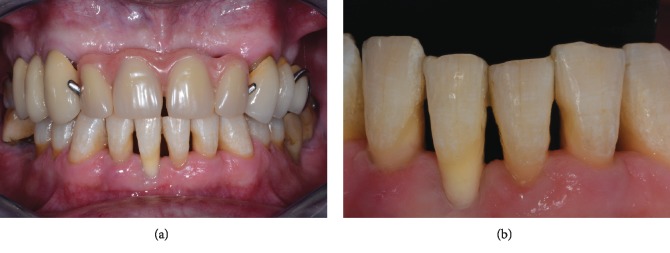
Postoperative functional and esthetic result at 6 months of follow-up: (a) full-mouth and (b) cross-polarized views of the completed restoration.

The patient was recalled six months later; no fractures of the splint or pontic failure (partial or full debonding) were recorded during this period. A satisfactory esthetic integration with adjacent lower incisors is shown (Figure 12).

## 3. Discussion

When facing a lost mandibular anterior tooth, alveolar defects of the edentulous area, and/or periodontal disease of adjacent teeth might be present, making implant-supported or traditional FPD restorations more difficult. Our case report demonstrated the chairside fabrication of an FRC-prosthesis, using a natural tooth as pontic, that solution represents a minimally invasive approach for tooth replacement. In addition, the glass fiber composite splint was simultaneously used for stabilization of adjacent teeth with reduced periodontal support [25]. According to current indications [26, 27], splinting of periodontally compromised mobile incisors is an option in case of advanced horizontal bone loss, to improve the patient's comfort (including speaking, biting, and chewing) and to provide better control of occlusion. Statistically significant changes of bone level at splinted teeth over a 10-year period were not observed when active and regular maintenance therapies were undertaken [27]; this way, splinting (that in our case was associated with tooth replacement) can be considered an adjunctive measure to maintain periodontally compromised mobile anterior mandibular teeth in patients attending regular supportive care [27].

Li et al. evaluated indirect (lab fabricated) glass fiber-reinforced RBFPD as periodontal splints to replace lost anterior teeth: a survival rate of 89% at the fourth year was found; the bleeding index scores and probing depths (mm) of adjacent teeth significantly improved from 1 year after the restoration to the end of the observation period [25]. A chairside direct prosthesis fabricated using the patient's own natural tooth might offer an economical advantage when looking at clinical/laboratory costs and number of appointments: in contrast to the development of classic RBFPDs, like those described by Li et al. [25], traditional impressions and working models are avoided, and time for indirect fabrication is saved.

Few data is available for the long-term survival/success of natural tooth pontic prosthesis. The mean follow-up period of published case reports on that topic is 1 year [19, 20]; however, other authors demonstrated excellent functional and esthetic results for longer time intervals, at two- [18] and six-year [17] follow-ups, for example. Quirynen et al. carried out a long-term evaluation of composite-bonded natural teeth as replacement of lower incisors with terminal periodontitis [28]: they found a survival rate of 80% after 5 years of function: the abutment teeth also showed stable probing depths and a negligible loss in attachment (0.1 mm/year). Among other factors, the improved condition of abutment teeth might also be explained by a supragingival placement of prosthetic or composite margins (with traditional RBFPD or natural tooth pontic prosthesis, respectively); in fact, both oral hygiene procedures and long-term maintenance of the restoration are facilitated. According to Sconnenschein et al., the probing depth of splinted mandibular teeth decreased from 3.39 mm to 2.12 mm and remained stable over the 3-year observation period, with the application of a strict supportive periodontal therapy; no splinted tooth was lost within the first 3 years after splinting [27].

A single-retainer, cantilever design has been suggested for traditional and all-ceramic anterior RBFPDs [8]; in fact, a recent systematic review has highlighted a significant lower survival rate for two-retainer (inlay or surface-only) restorations [9]. Single-retainer RBFPDs might reduce the risk of fracture of the adhesive cement (debonding), induced by unsynchronized movement of the abutment teeth in different directions under functional load [9, 29]. Despite a cantilevered, resin-bonded bridge with a natural tooth pontic might be planned, an optimal periodontal condition of the abutment tooth is recommended [29]; for this reason, a cantilever design was not selected for our patient. Further contraindications for RBFPDs, regardless an artificial or natural pontic, are represented by limited interocclusal space (deep bite), low overjet, parafunctional habits, and short clinical crowns [29]: recurrent debonding or fractures may occur with that unfavourable conditions.

The natural tooth pontic might be seated in a number of ways: flow or restorative composite without any additional reinforcement [18], metal wires, or fiber materials combined with resin composite. A splint made of stainless steel wire (round, twisted, or multistranded) and composite represents an alternative fixation method of the extracted tooth to adjacent elements and also for periodontal stabilization [30, 31]. Splints consisting of metal wire and composite resin have an interface with different elastic moduli and may be more susceptible to fatigue failure initiation [32]. According to Foek et al., specimens of the braided stainless steel wire group frequently failed at the composite-wire interface; on the other hand, the debonding forces were statistically similar to the nonmetal retainers [33]. Ribbond® retainers presented adhesive failure and material breakage in 50% and 40% of the specimens, respectively [33]. Resin adhesion to polyethylene FRCs might be less favorable because of the difficulty in plasma coating, silanization, and impregnation of the polyethylene fibers; such combinations of failure types may not cause direct enamel damage but will necessitate removal of the attached retainers and renewal of the bonding procedure [33]. Insights regarding the biomechanical behavior of teeth splinted using different materials have been also reported: according to Soares et al., periodontal splints with composite and adhesive systems were more effective than a rigid fixation method (stainless steel wire) in reducing the mandibular bone strain levels during occlusal function [32]. According to Sfondrini et al., the maximum load resistance of full-bonded fiber-reinforced composite splint is higher than a fixation obtained using metal wires combined with traditional composite [34]: despite an optimal splinting material has not been identified yet, to improve the mechanical strength of our direct prosthesis, a ribbon-based splint was chosen. Inlay-like retentions, slots, or groove preparation of abutment teeth is possible. Despite a slot-type preparation (or its size) of abutment teeth might have limited influence on the strength of the bonded bridge [35], that specific design was selected for two main reasons: (1) coverage of the fiber with an increased amount of composite, to avoid long-term exposure of fiber itself, and (2) patient's comfort, in order to minimize the thickness of incisors' surfaces at the lingual side.

Known limitations of resin-bonded prosthesis (including a natural pontic, as in our case) are related to fractures of the framework/splint, exposures of the fiber, and partial/full debonding of the attached tooth; long-term wear, discoloration, microleakage, or secondary caries at the tooth-restorative interface are also possible. Despite common adhesive techniques can be used for intraoral repairings or reluting, these complications imply additional interventions (repairings or substitution), costs, and sometimes unscheduled appointments at the office. According to Graetz et al., 75.3% of splinted teeth in periodontitis patients required repair (from zero up to three repairs/splint/year), highlighting potential frequent interventions in the long term [31]. Next studies should be focused on long-term evaluations of natural tooth pontic prosthesis; the soft and hard tissues behavior or modifications under the pontic site should be further clarified. Current ridge preservation techniques using biomaterials, membranes, or a partial extraction approach could be enhanced by the immediate insertion of a natural or artificial tooth pontic [36]: new insights are necessary when a combination of these procedures is adopted.

Despite the limitations of our study, an excellent functional, esthetic, mid-term result has been achieved adopting a minimally invasive restorative solution for single-tooth replacement; whether indicated or necessary, the natural tooth pontic technique leaves open other treatment options for the future.
